# Delineation and Diagnostic Criteria of Oral-Facial-Digital Syndrome Type VI

**DOI:** 10.1186/1750-1172-7-4

**Published:** 2012-01-11

**Authors:** Andrea Poretti, Giuseppina Vitiello, Raoul CM Hennekam, Filippo Arrigoni, Enrico Bertini, Renato Borgatti, Francesco Brancati, Stefano D'Arrigo, Francesca Faravelli, Lucio Giordano, Thierry AGM Huisman, Miriam Iannicelli, Gerhard Kluger, Marten Kyllerman, Magnus Landgren, Melissa M Lees, Lorenzo Pinelli, Romina Romaniello, Ianina Scheer, Christoph E Schwarz, Ronen Spiegel, Daniel Tibussek, Enza Maria Valente, Eugen Boltshauser

**Affiliations:** 1 Department of Pediatric Neurology, University Children's Hospital of Zurich, Switzerland; 2 Division of Pediatric Radiology, Russell H. Morgan Department of Radiology and Radiological Science, The Johns Hopkins University School of Medicine, Baltimore, MD, USA; 3 Mendel Laboratory, IRCCS Casa Sollievo della Sofferenza Institute, San Giovanni Rotondo, Italy; 4 Department of Pediatrics, Academic Medical Centre, Amsterdam, The Netherlands; 5 Department of Neuroradiology, Scientific Institute 'E. Medea', Bosisio Parini (LC), Italy; 6 Unit of Molecular Medicine, Department of Neurosciences, Bambino Gesù Hospital, Rome, Italy; 7 Department of Neurorehabilitation 1, Scientific Institute 'E. Medea', Bosisio Parini (LC), Italy; 8 Department of Biomedical Sciences, Ce.S.I. Aging Research Center, Gabriele d'Annunzio University Foundation, Chieti, Italy; 9 Department of Developmental Neurology, IRCCS Istituto Neurologico C. Besta, Milan, Italy; 10 Department of Medical Genetics, Ospedale Galliera, Genoa, Italy; 11 Division of Child Neurology, Spedali Civili, Brescia, Italy; 12 Division of Pediatric Neurology, Epilepsy Center, Vogtareuth, Germany; 13 Paracelsus Medical University, Salzburg, Austria; 14 Department of Neuropaediatrics, The Queen Silvia Children's Hospital, Sahlgrenska University, Gothenberg, Sweden; 15 Department of Pediatrics, Developmental Neurology, Skaraborg Hospital, Skövde, Sweden; 16 Clinical Genetics Unit, Great Ormond Street Hospital for Children, London, UK; 17 Division of Neuroradiology, Spedali Civili, Brescia, Italy; 18 Division of Diagnostic Imaging, University Children's Hospital of Zurich, Switzerland; 19 Department of Neonatology, University Children's Hospital, Tübingen, Germany; 20 Department of Pediatrics A, HaEmek Medical Center, Afula and Rappaport School of Medicine, Haifa, Israel; 21 Department of General Pediatrics, University Children's Hospital, Düsseldorf, Germany; 22 Department of Medical and Surgical Pediatric Sciences, University of Messina, Messina, Italy

**Keywords:** Joubert syndrome and related disorders, Oral-facial-digital syndrome type VI, neuroimaging; molar tooth sign, cerebellar malformation

## Abstract

Oral-Facial-Digital Syndrome type VI (OFD VI) represents a rare phenotypic subtype of Joubert syndrome and related disorders (JSRD). In the original report polydactyly, oral findings, intellectual disability, and absence of the cerebellar vermis at post-mortem characterized the syndrome. Subsequently, the molar tooth sign (MTS) has been found in patients with OFD VI, prompting the inclusion of OFD VI in JSRD. We studied the clinical, neurodevelopmental, neuroimaging, and genetic findings in a cohort of 16 patients with OFD VI. We derived the following inclusion criteria from the literature: 1) MTS and one oral finding and polydactyly, or 2) MTS and more than one typical oral finding. The OFD VI neuroimaging pattern was found to be more severe than in other JSRD subgroups and includes severe hypoplasia of the cerebellar vermis, hypoplastic and dysplastic cerebellar hemispheres, marked enlargement of the posterior fossa, increased retrocerebellar collection of cerebrospinal fluid, abnormal brainstem, and frequently supratentorial abnormalities that occasionally include characteristic hypothalamic hamartomas. Additionally, two new JSRD neuroimaging findings (ascending superior cerebellar peduncles and fused thalami) have been identified. Tongue hamartomas, additional frenula, upper lip notch, and mesoaxial polydactyly are specific findings in OFD VI, while cleft lip/palate and other types of polydactyly of hands and feet are not specific. Involvement of other organs may include ocular findings, particularly colobomas. The majority of the patients have absent motor development and profound cognitive impairment. In OFD VI, normal cognitive functions are possible, but exceptional. Sequencing of known JSRD genes in most patients failed to detect pathogenetic mutations, therefore the genetic basis of OFD VI remains unknown. Compared with other JSRD subgroups, the neurological findings and impairment of motor development and cognitive functions in OFD VI are significantly worse, suggesting a correlation with the more severe neuroimaging findings. Based on the literature and this study we suggest as diagnostic criteria for OFD VI: MTS and one or more of the following: 1) tongue hamartoma(s) and/or additional frenula and/or upper lip notch; 2) mesoaxial polydactyly of one or more hands or feet; 3) hypothalamic hamartoma.

## Introduction

Joubert Syndrome (JS) is a rare midbrain-hindbrain malformation with an estimated prevalence between 1:80 000 and 1:100 000 live births [1]. The characteristic neurological signs of JS include muscular hypotonia (most prominent during infancy), cerebellar ataxia (typically developing later), ocular motor apraxia, and an irregular breathing pattern in the neonatal period [2-4]. Additionally, cognitive functions are impaired in almost all patients [5]. The so-called molar tooth sign (MTS) is a consistent and pathognomonic neuroanatomical feature of JS [6-9]. The MTS is characterized by thickened, elongated, and horizontally located superior cerebellar peduncles (SCP) and an abnormally deep interpeduncular fossa. Involvement of the kidneys (nephronophthisis and/or renal cysts), liver (congenital liver fibrosis), and eyes (retinal dystrophy and/or ocular colobomas) may be associated features of JS, defining the spectrum of so-called JS-related disorders (JSRD) [2-4]. At present, causative mutations in 13 genes have been associated with JSRD (JBTS1/*INPP5E*, JBTS2/*TMEM216*, JBTS3/*AHI1*, JBTS4/*NPHP1*, JBTS5/*CEP290*, JBTS6/*TMEM67*, JBTS7/*RPGRIP1L*, JBTS8/*ARL13B*, JBTS9/*CC2D2A*, JBTS10/*OFD1*, JBTS12/*KIF7*, JBTS13/*TCTN1 *and the *TCTN2 *gene) [4,10-12]. Additionally, heterozygous, not causative mutations have been found in the JBTS11/*TTC21B *gene [13]. All JSRD genes encode for proteins of the primary cilium. Primary cilia are subcellular organelles known to play key roles in the development and functioning of several cell types, including retinal photoreceptors, neurons, and the epithelial cells lining kidney tubules and bile ducts [14-16].

Based on the extent of multiorgan involvement, six phenotypes of the JSRD spectrum have been recently defined: 1) *"pure" JS *(purely neurological without retinal, renal, or liver involvement); 2) *JS with ocular defect *(neurological features associated with retinal dystrophy); 3) *JS with renal defect *(neurological features with renal involvement, mostly nephronophthisis); 4) *JS with oculorenal defects *(association of neurological signs with both retinal dystrophy and nephronophthisis); 5) *JS with hepatic defect *(neurological features with congenital liver fibrosis); and 6) *JS with oral-facial-digital defects *[4].

The sixth JSRD phenotype represents the Oral-Facial-Digital Syndrome type VI (OFD VI) or Váradi-Papp syndrome (OMIM 277170). The original description in the pre-MRI era included polydactyly, oral findings, intellectual disability, and absence of the cerebellar vermis at post-mortem [17]. Subsequently, MTS has been found in patients with OFD VI prompting the inclusion of OFD VI in JSRD [8,18]. Additionally, hypothalamic hamartomas were reported as a characteristic, but not consistent part of OFD VI [8,18-20]. Absence of the pituitary gland was described in two siblings [8,18-20]. To date no diagnostic criteria for OFD VI have been proposed.

Based on the largest cohort of OFD VI patients collected so far, we aimed to: 1) evaluate the spectrum of neuroimaging findings, 2) characterize the neurological and dysmorphic features, the involvement of other organs, and the neurodevelopmental outcome, 3) describe the results of genetic screening, and 4) suggest diagnostic criteria.

## Patients and Methods

### Patient cohort

The patients included in this study were collected by the senior authors (EMV and EB): 1) from their personal cohorts of OFD VI patients; 2) from patients who were referred for second opinion; 3) from requests to evaluate clinical and neuroimaging data of patients with a definite or probable diagnosis of OFD VI; and 4) from patients referred for molecular genetic testing after a diagnosis of OFD VI. The inclusion criteria were:

1. MTS and one oral finding (one or multiple tongue hamartomas, or multiple prominent frenula, or cleft lip/palate) and polydactyly (pre-, meso-, or postaxial), or

2. MTS and more than one typical oral finding.

Inclusion criterion 2 was derived from the literature suggesting that oral findings are consistently present in and diagnostic for OFD VI in the context of JSRD.

### Neuroimaging analysis

All available imaging data sets were retrospectively studied and evaluated by two pediatric neurologists with experience in JSRD (AP and EB). The same evaluation approach was adopted as in the recent study of neuroimaging findings in 75 patients with JSRD [21].

#### Infratentorial image analysis

The infratentorial evaluation included qualitative assessment of the size (degree of hypoplasia) and morphology (normal or folial disorganization) of the *cerebellar vermis *and *hemispheres *(judged as normal, reduced, or enlarged by visual evaluation). The extent of vermian hypoplasia was graded according to Quisling et al [7].

On sagittal images the size and shape of the *fourth ventricle *was evaluated. On axial images the width of the *interhemispheric cleft *was measured and graded ("slit" if narrower than 1 mm, 1-2 mm, or wider than 2 mm). The *posterior fossa *was qualitatively evaluated on sagittal images, including size (normal or enlarged), presence of *increased retrocerebellar cerebrospinal fluid (CSF) collection *(best assessed on axial images, however), and size of the *prepontine cistern *(normal, or enlarged if wider than twice the cross-sectional diameter of the basilar artery).

The *superior cerebellar peduncles *(*SCP*) were evaluated on axial images for width (minimally or obviously thickened), length (normal, or very elongated if almost equally as long as the posterior fossa), symmetry (symmetric or asymmetric), morphology (smooth or irregular contour), and axial orientation (parallel, A-like or divergent course, V-like or convergent course, or curved) as well as sagittal orientation (horizontal as typically in JSRD or ascending when the angle between the brainstem and SCP is greater than 90°). The size of the *interpeduncular fossa *was assessed and graded as minimally or obviously deepened. The cerebral peduncles forming the *"crown" of the molar tooth *were evaluated for asymmetry on axial images.

The *brainstem *was assessed on sagittal images. The *mesencephalon *was evaluated regarding morphology (normal or abnormal), length (normal, shortened, or elongated), and width (normal, thinned, or thickened). The presence of pre-mesencephalic grey matter heterotopias lying within the interpeduncular fossa was ascertained. The morphology of the *tectum, pons*, and *medulla *(normal or abnormal) as well as the width of pons and medulla (normal, reduced, or enlarged) were studied.

*Cephaloceles *in two locations were evaluated: occipitally and at the level of the foramen magnum.

#### Supratentorial image analysis

The supratentorial evaluation included a search for *migrational disorders *(particularly polymicrogyria and heterotopias), *midline defects *(dysgenesis of the corpus callosum and absence of the septum pellucidum), *hippocampal malrotation, ventriculomegaly *(as an enlargement of the lateral ventricles without signs of increased intracranial pressure), *hypothalamic hamartoma, absence of the pituitary gland, fusion of the thalami*, presence of a *cavum Vergae *and *temporal lobe hypoplasia*, and *white matter signal abnormalities *(focal or diffuse).

### Clinical analysis

Detailed information about neurological and dysmorphic features, involvement of other organs, and neurodevelopmental/neurocognitive outcomes were provided by review of the clinical history and updated clinical-neurological follow-up examination.

The neurological features assessed included truncal and limb ataxia, muscular hypotonia, ocular motor apraxia, nystagmus, and strabismus. Additionally, we searched for a history of neonatal breathing abnormalities and epileptic seizures. We characterized the type of oral findings (tongue hamartoma(s), multiple prominent frenula, cleft lip and/or palate, and upper lip notch) and polydactyly (pre-, meso-, or postaxial as duplication of the first, second to fourth, and fifth digit or ray, respectively) to report their frequency. We also assessed typical JSRD craniofacial dysmorphic features such as broad nasal tip, frontal bossing, hypertelorism, and ptosis.

Associated involvement of other organs was explored with particular attention to the kidneys (nephronophthisis and cystic dysplastic kidneys), eyes (retinal dystrophy, congenital retinal blindness, and uni- or bilateral colobomas of the iris, choroidea, or retina), and liver (congenital liver fibrosis resulting from ductal plate malformation).

Finally, we evaluated the cognitive, motor, and language outcomes. If possible, formal neuropsychological testing with assessment of the intelligence quotient (IQ) was performed. Otherwise, the developmental stage was estimated from the patient's history, clinical observations, and kindergarten or school reports.

### Genetic studies

Most patients included in this study had undergone mutation analysis of some JSRD causative genes as part of previous genetic screenings on large JSRD cohorts [22-28] or in subsequent research studies, following the same mutation screening protocols. The two siblings from consanguineous parents (patients 12 and 13, Table 1) underwent genome-wide homozygosity mapping using a GeneChip SNP-array 6.0 platform (Affymetrix, Santa Clara, CA, USA), as previously reported [29].

**Table 1:** Key clinical and neuroimaging findings in 16 patients with OFD VI

Patients	Age at last follow-up (years)	Oral findings				Polydactyly				MTS	Hypothalamic hamartoma	Additional features
					
		Tongue hamartomas	Multiple frenula	Cleft lip/palate	Upper lip notch	Hand right	Hand left	Foot right	Foot left			
1	28.4	+	-	-	-	Preaxial	Preaxial	Preaxial	Preaxial	+	-	Dysplastic kidney

2	0.2°	-	+	-	-	Postaxial	Postaxial	-	-	+	+	Morning glory anomaly

3	4.4	+	+	-	-	Postaxial	Postaxial	Preaxial	Preaxial	+	+	ASD, short stature (no endocrine dysfunctions)

4	5.5	-	-	+	-	Postaxial	Postaxial	Postaxial	Postaxial	+	-	Retinal colobomas

5	1.8°	+	-	+	-	-	-	-	-	+	-	-

6*	16.5	+	-	-	-	-	Postaxial	-	Postaxial	+	-	Choroidal colobomas, optic atrophy

7*	16.5	+	-	+	-	Postaxial	Postaxial	-	-	+	-	Choroidal colobomas, optic atrophy

8	0.6	+	-	-	-	Postaxial	Postaxial	Postaxial	Postaxial	+	-	-

9	7.1	+	-	-	-	Postaxial	Postaxial	-	-	+	-	High grade myopia

10	22.4	+	-	-	-	Postaxial	-	Preaxial	Preaxial	+	-	-

11	9.3	+	+	-	+	Mesaxial	Preaxial	Preaxial	Preaxial	+	+	Hirschsprung disease

12**	17.9	-	-	+	-	-	Postaxial	-	-	+	-	Choroidal and retinal colobomas, LCA, NPHP

13**	10.0	+	-	-	-	Postaxial	Postaxial	Postaxial	Postaxial	n.a.	n.a.	Choroidal and retinal colobomas, LCA

14	17.0	+	-	-	-	-	-	Preaxial	Preaxial	+	-	-

15	17.7	+	-	+	+	Mesaxial	Mesaxial	Preaxial	Preaxial	+	+	Bicuspid aortic valve

16	2.0	+	+	-	+	Postaxial	Postaxial	Preaxial	Preaxial	+	-	-

ASD, Atrial septal defect; LCA, Leber's congenital amaurosis; MTS, Molar tooth sign; n.a., not applicable; NPHP, nephronophthisis; OFD VI, Oral-Facial-Digital Syndrome type VI; *, Twins with heterozygous c.517G > A mutation within the AHI1 gene; **, Siblings; °, died.

## Results

### Patient characteristics

Fifteen patients fulfilled the inclusion criteria. Additionally, the brother of patient 12 has been included, presenting with tongue hamartomas, postaxial polydactyly of all limbs, bilateral choroidal and retinal colobomas, severe cognitive impairment, and similar craniofacial phenotype, although no MRI was performed. Overall, 16 patients could be included in the study (11 males and 5 females, Table 1). Our cohort included two siblings and one pair of twins, from different families. The patients originated from Italy (n = 5), Germany (n = 3), Israel (n = 2), Switzerland (n = 2), Bosnia (n = 1), Cyprus (n = 1), Sweden (n = 1), and Turkey (n = 1). Parental consanguinity was observed in two families (patients 4, 12, and 13). Neuroimaging findings of patients 1, 2, and 6 have been included in previous reports [21]. At the last follow-up at the median age of 10.5 years (mean 11.5 years, range 2.5 months to 28.4 years), 14 patients were alive. One patient died at the age of 2.5 months due to severe apneas that necessitated repeated resuscitation; another patient died at the age of 1.8 years due to severe pneumonia and septic shock. Postmortem examinations were not performed.

### Neuroimaging

MRIs of 15 patients were available for evaluation. All patients underwent MRI at their local hospital for clinical indications. At the time of the MRIs, the median age of the patients was 2.4 years (mean 6.8, range 2 days to 22.2 years). In all cases the MRI examinations included at least axial and sagittal T1- and T2-weighted MRI.

### Infratentorial findings

**Figure 1:**
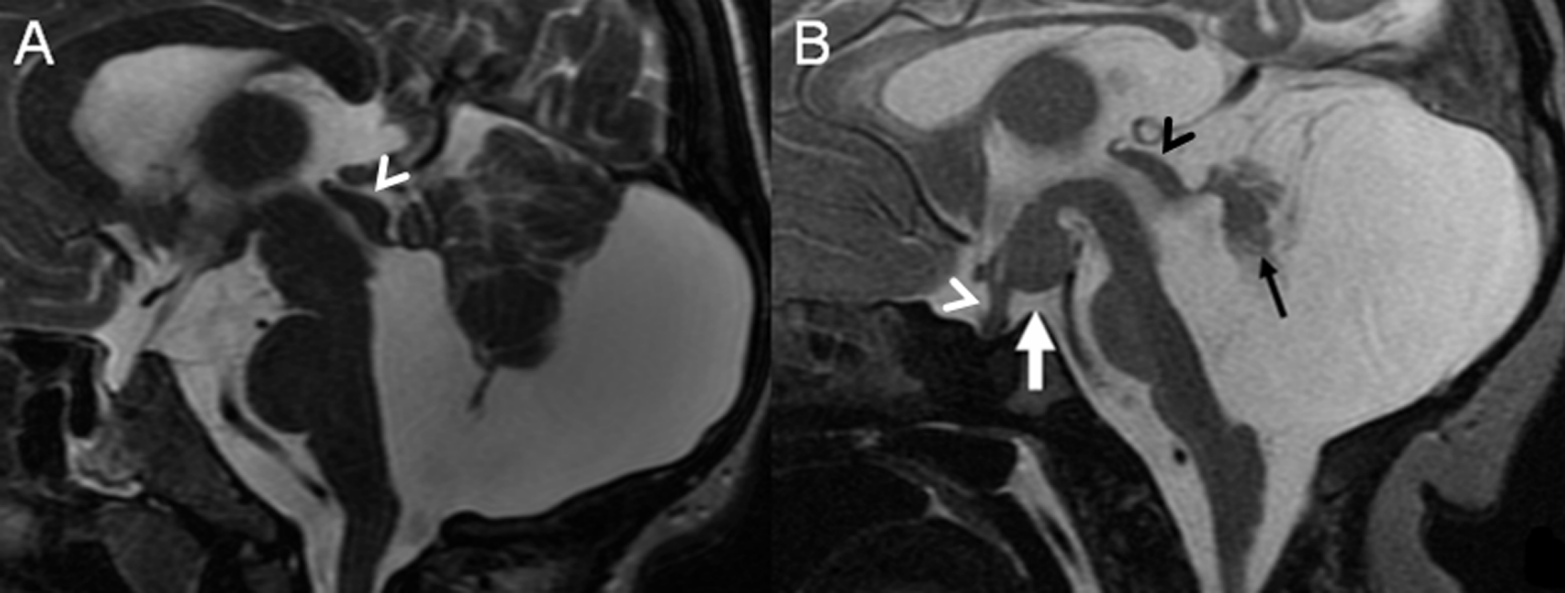
**Midsagittal T2-weighted MR images of a 22-year-old woman (A) and a 2-day-old neonate (B), modified from Poretti A et al, AJNR, 2008, with permission) with OFD VI reveal an enlarged posterior fossa with marked retrocerebellar CSF collection**. Additionally, in both patients the brainstem appears dysmorphic: in A the midbrain is thickened, the tectum dysplastic (white arrow head), and the pons short; in B there is elongation of the mesencephalon, reduced size of the pons, and dysplasia of the tectum (black arrow head). In both patients the cerebellar vermis is hypoplastic and its remnants are dysplastic (black arrow in B), the massa intermedia is prominent, and in B a hypothalamic hamartoma is seen (white arrow) and the pituitary stalk appears thickened (white arrow head).

Infratentorial neuroimaging findings are summarized in Table 2. The *hypoplasia of the cerebellar vermis *was severe in ten patients (67%) and moderate in five (33%) (Figure 1). The *vermian remnants *appeared dysplastic in all patients (Figure 1). The *volume of the cerebellar hemispheres *was reduced in six patients (40%) and their *folial organization *was abnormal in eight (53%). The *size of the posterior fossa *was enlarged in ten patients (67%) (Figure 1) and in seven patients (47%) an increased amount of *retrocerebellar CSF collection *was present.

**Table 2:** Infratentorial neuroimaging findings in 15 patients with OFD VI

Cerebellar and brainstem neuroimaging findings			Patients (%)
Posterior fossa	Size	Enlarged	10 (67%)
	
	Retrocerebellar fluid	Marked	7 (47%)
	
	Prepontine cistern	Enlarged	4 (27%)
	
	Foramen magnum cephalocele		2 (13%)

Cerebellar hemispheres	Size	Reduced	6 (40%)
		Enlarged	0 (0%)
	
	Morphology	Folial disorganization	8 (53%)
	
	Interhemispheric cleft	< 1 mm	8 (53%)
		1-2 mm	4 (27%)
		> 2 mm	3 (20%)

Superior cerebellar peduncles	Width	Minimally thickened	4 (27%)
		Obviously thickened	11 (73%)
	
	Length	Very elongated	1 (7%)
	
	Axial orientation	Parallel	10 (67%)
		A-like	2 (13%)
		V-like	0 (0%)
		Curved	3 (20%)
	
	Sagittal orientation	Horizontal	7 (47%)
		Ascending	8 (53%)
	
	Morphology	Irregular contour	1 (7%)

Ponto-mesencephalic isthmus	Interpeduncular fossa	Obviously deepened	14 (93%)
		Minimally deepened	1 (7%)
	
	Interpeduncular heterotopia		2 (13%)

Cerebral peduncles	Tooth-crown	Asymmetric	3 (20%)

Mesencephalon	Morphology	Dysmorphic	2 (13%)
	
	Length	Shortened	0 (0%)
		Elongated	2 (13%)
	
	Width	Thinned	3 (20%)
		Thickened	4 (27%)
	
	Tectum	Dysmorphic	10 (67%)

Pons	Width	Reduced	5 (33%)
		Enlarged	0 (0%)

Medulla	Morphology	Dysmorphic	1 (7%)
	
	Width	Reduced	0 (0%)
		Enlarged	1 (7%)

OFD VI, Oral-Facial-Digital Syndrome type VI

**Figure 2:**
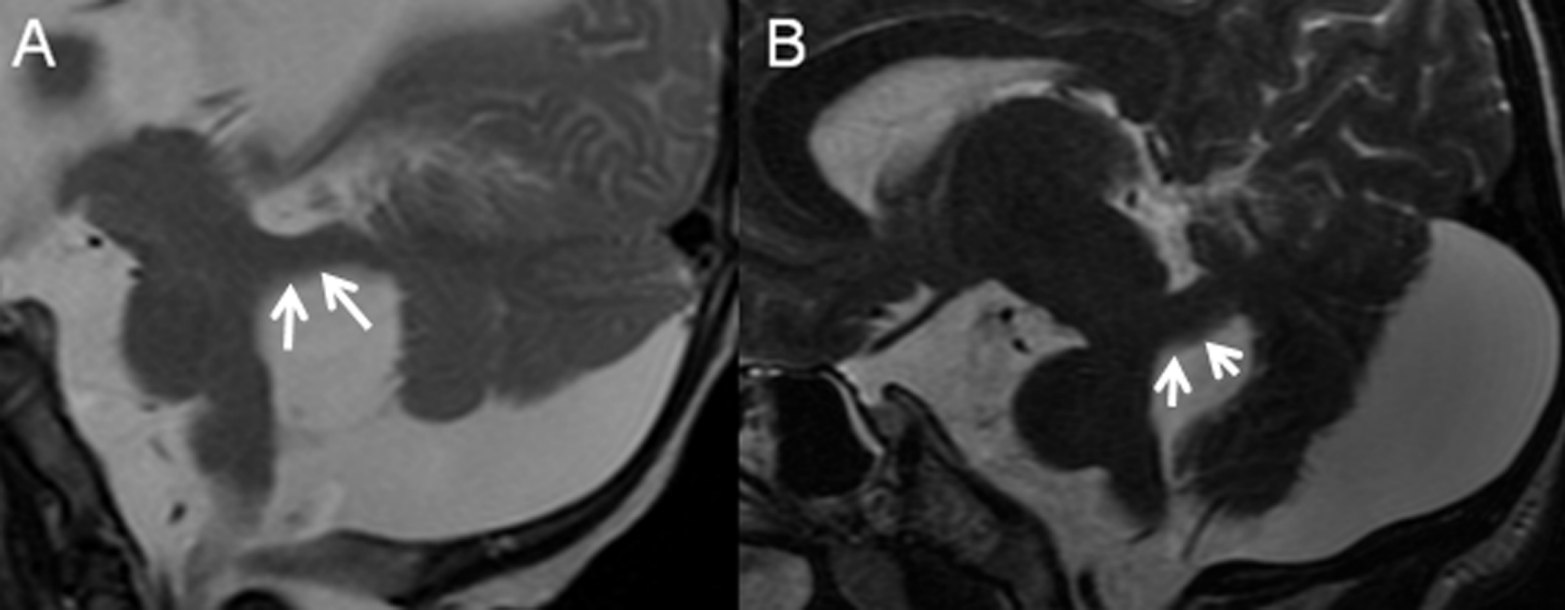
**Parasagittal T2-weighted MR images of a 5-month-old infant (A) and a 22-year-old woman (B) with OFD VI**. In JSRD the SCP (white arrows) have a characteristic horizontal sagittal orientation (A). In some patients with OFD VI, however, the SCP (white arrows) have a clearly ascending orientation (B).

The *SCP *were asymmetric in five patients (33%) and in one patient their contour was irregular, showing some notches. The *axial orientation of the SCP *was variable, the *sagittal orientation *was horizontal in seven patients (47%) and ascending in eight (53%) (Figure 2). The *cerebral peduncles *were asymmetric in three patients (20%) giving them the appearance of a decaying molar tooth on axial images.

**Figure 3:**
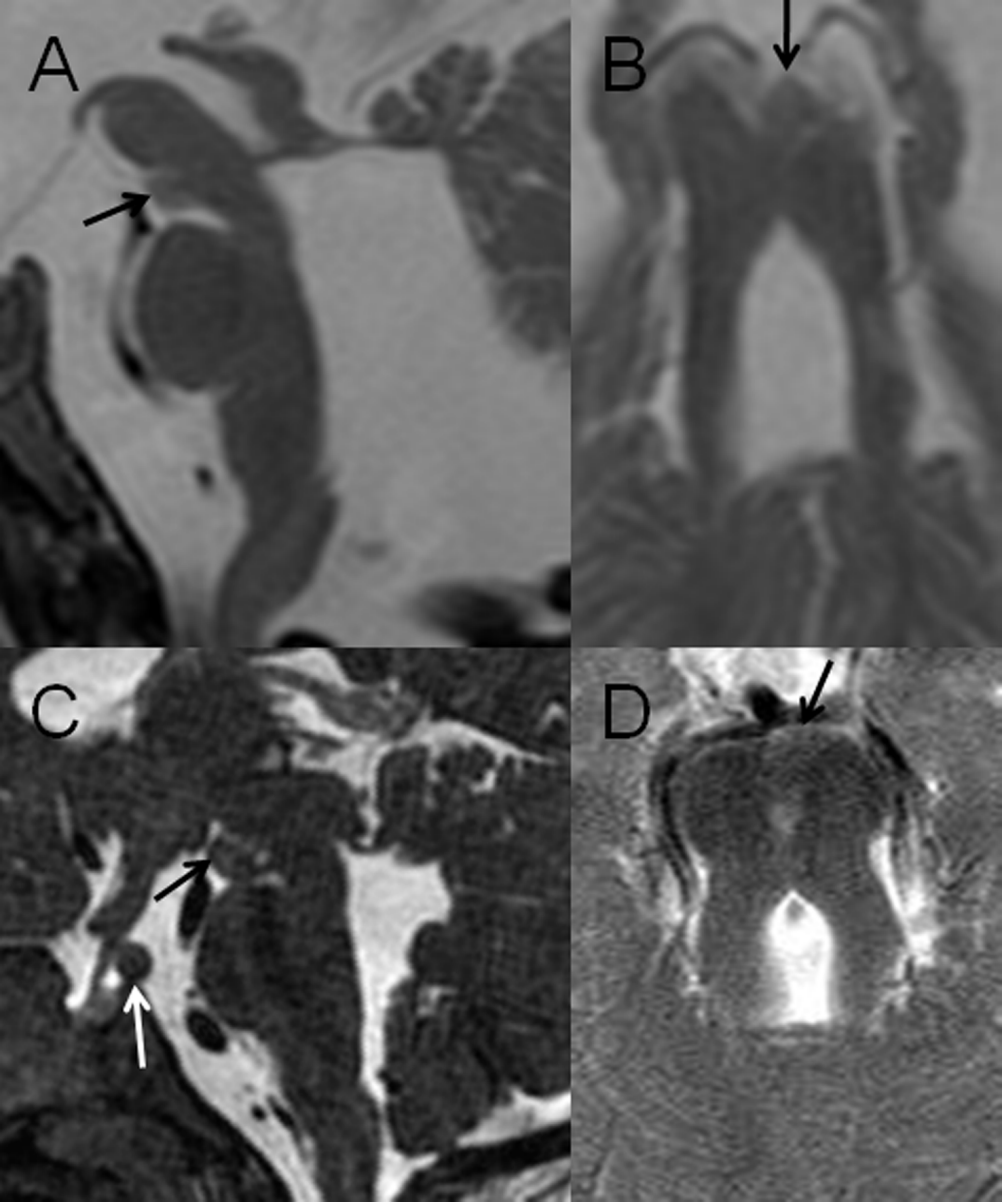
**A, midsagittal and B, axial T2-weighted MR images of a 5-month-old infant with OFD VI, and C, midsagittal constructive interference in steady-state (CISS) and D, axial T2-weighted MR images of a 2-year old child with OFD VI**. In both patients there is a grey matter isointense, nodular tissue mass in the interpeduncular fossa representing an interpeduncular heterotopia (black arrows). Additionally, C demonstrates a hypothalamic hamartoma (white arrow) and a horizontal septum at the distal end of the Sylvian aqueduct is noted on A.

Abnormalities in size and/or shape of the *brainstem *were found in eleven patients (73%) (Figure 1). In two patients (13%) a pre-mesencephalic heterotopia was present (Figure 3). Two different locations of cephaloceles (occipitally and at the level of the foramen magnum) were distinguished.

### Supratentorial findings

**Figure 4:**
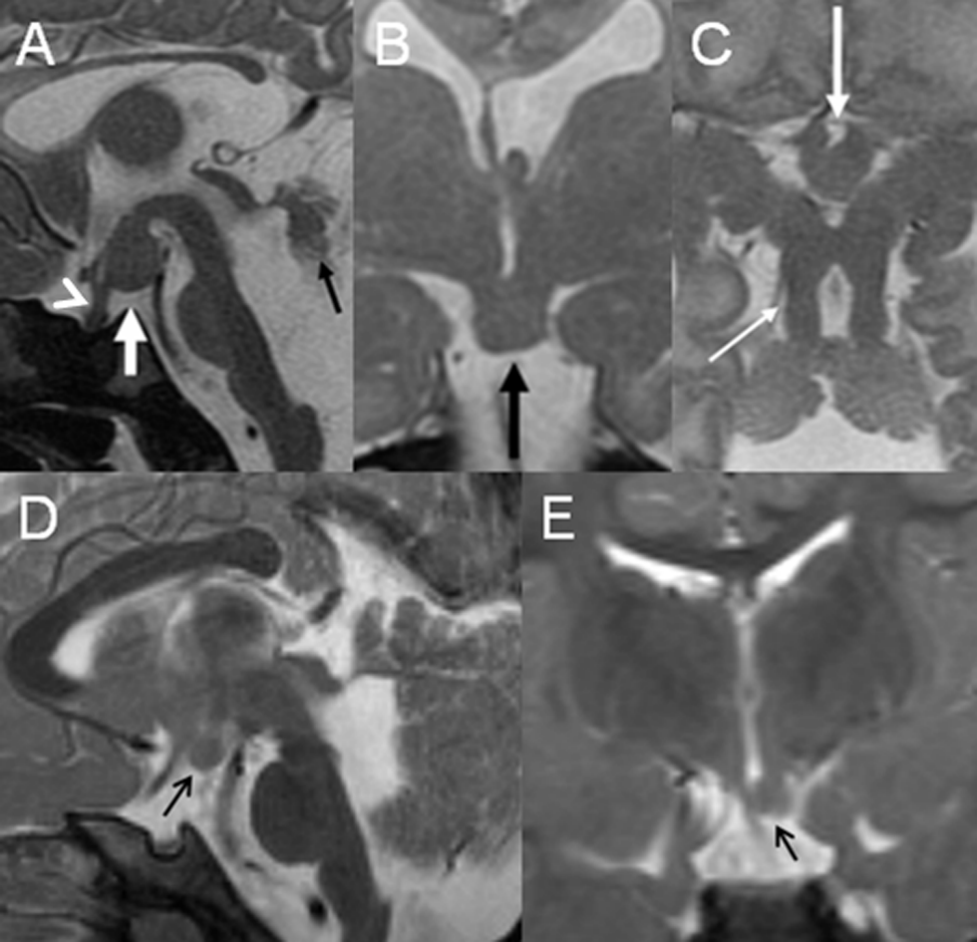
**A, midsagittal, B, coronal, and C, axial T2-weighted MR image of a 2-day-old neonate with OFD VI show a hypothalamic hamartoma (white arrow in A, black arrow in B, and thick white arrow in C)**. Also shown are significant vermian hypoplasia and dysplasia (black arrow in A), enlarged fourth ventricle and posterior fossa, the characteristic MTS (thin white arrow in C), elongation of the mesencephalon, reduced size of the pons, thin corpus callosum, absent of the left leaf of the septum pellucidum, and a thickened pituitary stalk (white arrow head in A; modified from Poretti A et al, AJNR, 2008, with permission). D, midsagittal and E, coronal T2-weighted MR image of a 2.3-year-old boy with OFD VI show a left paramedian hypothalamic hamartoma (black arrow). Moreover, D shows hypoplasia and dysplasia of the cerebellar vermis and enlargement of the fourth ventricle.

Supratentorial neuroimaging findings are summarized in Table 3. Seven patients had *hippocampal malrotation *(47%). The *corpus callosum *was dysgenetic (but not absent) in four patients (27%), the *septum pellucidum *was not identified in three (20%). Observed *migrational disorders *included bilateral polymicrogyria in three patients (20%), unilateral closed-lip schizencephaly in two (13%), and periventricular nodular heterotopias in one (7%). A *ventriculomegaly *was present in six patients (40%) and in four the *thalami *appeared fused (27%). A *hypothalamic hamartoma *was identified in four patients (27%) (Figure 4). The pituitary gland could be identified in all patients.

**Table 3:** Supratentorial neuroimaging findings in 15 patients with OFD VI

Supratentorial neuroimaging findings	Patients (%)
Hippocampal malrotation	7 (47%)
Ventriculomegaly	6 (40%)
Callosal dysgenesis	4 (27%)
Fused thalami	4 (27%)
Hypothalamic hamartoma	4 (27%)
Absent septum pellucidum	3 (20%)
Polymicrogyria	3 (20%)
Schizencephaly	2 (13%)
Heterotopias (periventricular, subcortical)	1 (7%)
Occipital cephalocele	1 (7%)
White matter signal abnormality (diffuse or focal)	1 (7%)
Cavum Vergae	1 (7%)
Temporal lobe hypoplasia	1 (7%)

OFD VI, Oral-Facial-Digital Syndrome type VI

### Clinical findings

At the last follow-up, 12 of 16 patients (75%) had generalized muscular hypotonia. Based on age and motor developmental stage, truncal ataxia could be evaluated in only eight patients but was always present (8/8). Limb ataxia was assessable in five patients and was present in four (4/5). Ten patients demonstrated ocular motor apraxia (63%), nine had strabismus (56%; divergent in eight patients and convergent in one), and three had nystagmus (19%). Additionally, positive pyramidal signs were present in two patients (13%) and dyskinetic movements in one (6%). Three patients suffered from epileptic seizures and developed severe scoliosis, respectively (19%). Seven patients had a history of neonatal respiratory abnormalities consisting of alternating short episodes of apnea and hyperpnoea (44%).

**Figure 5:**
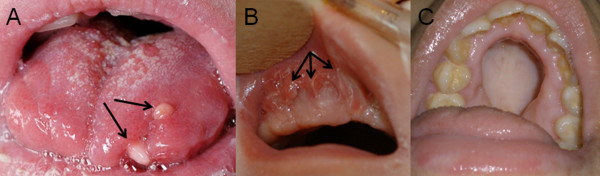
**A, tongue hamartomas (black arrows), B, additional prominent oral frenula (black arrows), and C, cleft palate in patients with OFD VI**.

**Figure 6:**
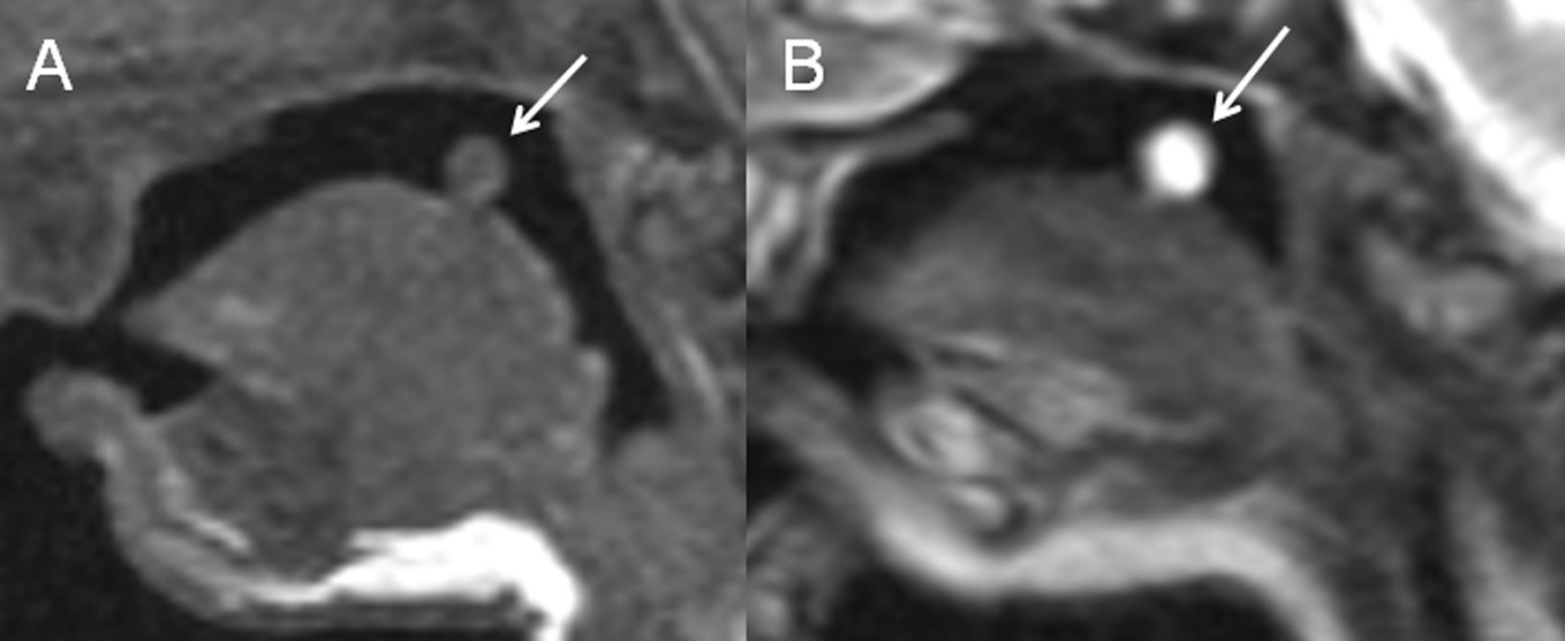
**A, sagittal T1-weighted and B, sagittal T2-weighted MR image of a 2-week-old newborn with OFD VI demonstrate a T1-isointense (white arrow on A) and T2-hyperintense (white arrow on B) tissue lesion on the surface of the posterior part of the tongue, representing a tongue hamartoma**.

**Figure 7:**
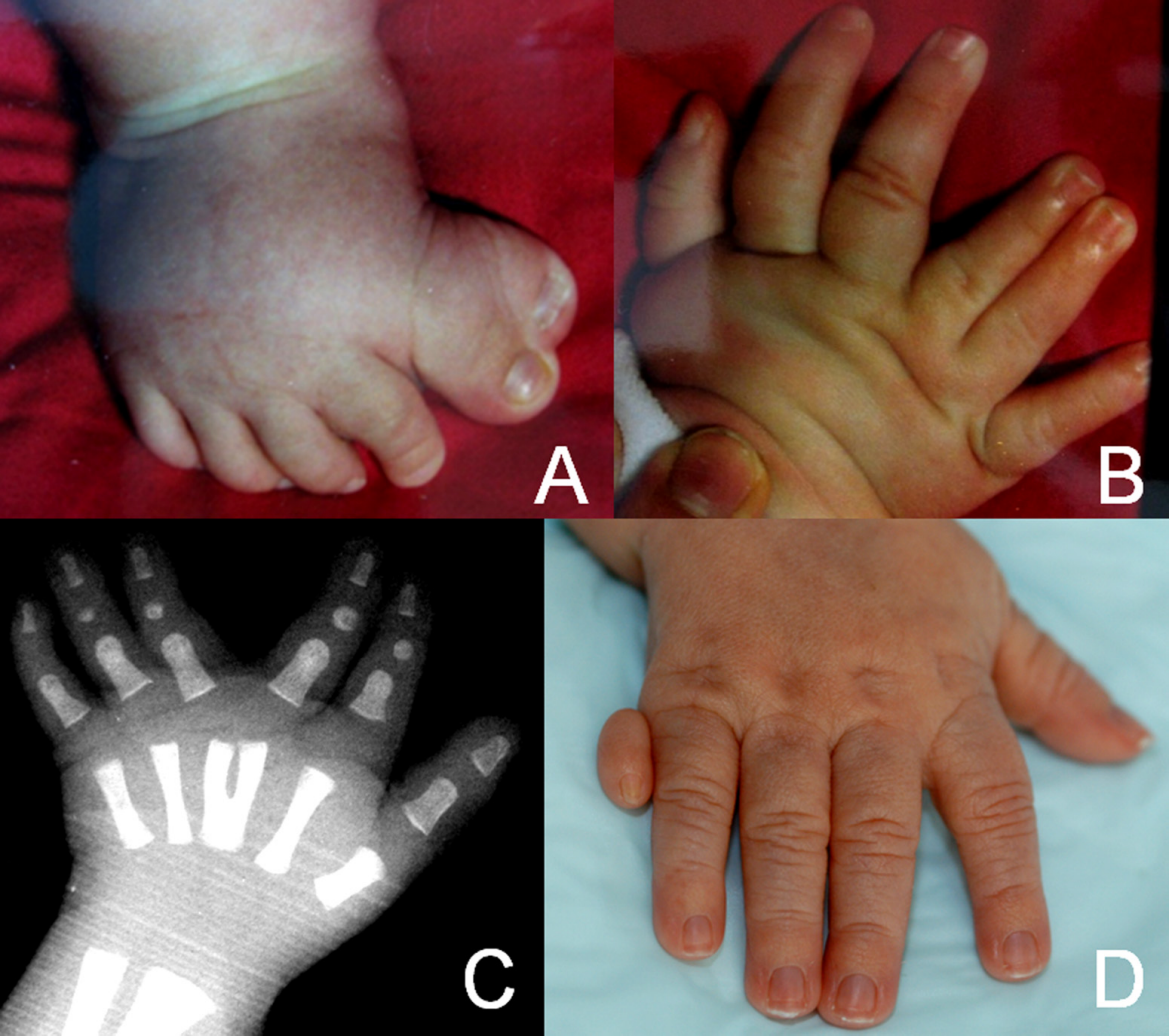
**A, preaxial poly-syndactyly of the right foot, B, mesoaxial polydactyly of the right hand, C, X-ray of the left hand showing a Y-shaped third metacarpal bone, and D, postaxial polydactyly of the right hand**.

Oral findings (Figures 5, 6) and polydactyly characteristics (Figure 7) are presented in Table 1. No patient had a lobed tongue. Craniofacial morphological manifestations included a broad nasal bridge in twelve patients, hypertelorism and frontal bossing in ten, and bilateral ptosis in five.

Involvement of other organs was found in eleven patients (69%, Table 1). Patient 1 had a dysplastic kidney with preserved renal function. Colobomas were present in five patients (31%), causing severe visual impairment in all. No patients had obvious endocrine dysfunctions based on assessment of growth parameters, external genitalia, and pubertal development.

Data on motor outcome were available for 15 patients. At the last follow-up, six patients could not sit or stand independently, two could sit and roll over but not stand or walk independently, whereas patient 9 did not show any motor development at the age of 7 months. Motor developmental delay was severe in three patients who started to walk at the age of 6-7 years, and mild in three other patients who started to walk at the age of 2 years.

Data about developmental or cognitive outcome were available for 15 patients, and were abnormal in 14 (93%). Based on clinical history and observations, nine patients had profound cognitive impairment with almost absent cognitive development. Patients 10 and 14 had a moderate learning disability with full-scale IQs of 58 and 47 respectively. The developmental quotient of patient 9 measured at the age of 73 months performing the Griffiths Mental Developmental Scale was 42, and patient 16 showed a global developmental delay at the age of 22 months. Patient 15 was the only one whose cognitive functions were within normal limits: full-scale IQ was 82, verbal IQ 94, and performance IQ 70 (tested by means of the revised Wechsler Adult Intelligence Scale (WAIS-R) at the age of 17.7 years). He attended a regular school and completed a vocational training course in graphic design. Additionally, neuropsychological assessment in three patients detected difficulties with spatial cognition including visual-spatial organization and memory, with impairment in all patients of executive functions such as planning, abstract reasoning, and working memory.

Speech and language could be evaluated in 14 of 16 patients based on their age, and all demonstrated some kind of speech or language impairment. Ten patients had no expressive speech and were only able to vocalize sounds. Three patients demonstrated dysarthria impairing speech, one also had severe language difficulties impairing syntax, and another patient did not develop speech, although his non-verbal, written development allowed him to communicate sufficiently. Additionally, orofacial dyspraxia was reported in three patients (19%), while three other patients had severe dysphagia necessitating a gastrostomy feeding tube.

### Genetic findings

DNA of 13 patients was available for genetic analysis. Pathogenic mutations in the following JSRD genes were excluded: *INPP5E *and *TMEM216 *(12 probands), *TMEM67 *and *RPGRIP1L *(10 probands), *CEP290 *(9 probands), *AHI1 *(4 probands). In both twins (patients 6 and 7) we found a heterozygous c.517G > A mutation in the AHI1 gene, which corresponded at the protein level to the p.A173T missense change. The homozygous *NPHP1 *deletion was excluded in all probands. Finally, genome-wide homozygosity mapping in the two siblings 12 and 13 allowed us to exclude linkage to all known JSRD loci. Mutation analysis of the *GLI3 *gene has not been performed in any patient.

## Discussion

The Oral-Facial-Digital Syndrome type VI (OFD VI) or Váradi-Papp syndrome was originally described as the combination of polydactyly (typically mesoaxial with Y-shaped metacarpals, but also pre- and postaxial), cleft lip/palate, lingual nodules, prominent oral frenula, and cognitive impairment in six children from a Hungarian gypsy colony [17]. The autopsy of one of these children showed absence of the cerebellar vermis. Subsequent reports confirmed the cerebellar anomalies as consistent findings and showed hypothalamic hamartomas as inconsistent features [19,30]. Al-Gazali et al were the first to report the MTS in two patients with OFD VI [18]. Gleeson et al included OFD VI as a subtype of JSRD and the MTS became a mandatory diagnostic criterion of OFD VI [8].

### Neuroimaging

In a recent study of neuroimaging in 75 patients with JSRD, we failed to show a specific correlation between neuroimaging and clinical phenotype or genotype in JSRD, but we noted that the four OFD VI patients demonstrated a similar and more severe neuroimaging pattern [21]. Indeed, this preliminary evidence was confirmed in the present study. An enlarged posterior fossa and a marked retrocerebellar CSF collection were present in about 70% and 50% of our OFD VI patients respectively, and were more frequent than in other JSRD subgroups. Moreover, in 40% of the OFD VI patients the cerebellar hemispheres appeared hypoplastic, while in other JSRD subgroups their size was mostly normal [7,21,31]. In OFD VI patients we found two additional MTS characteristics, not previously described: an ascending course and an irregular (dysplastic) contour of the SCP. Abnormalities in size and/or shape of the brainstem have been found in about 70% of the OFD VI patients, far more commonly than in the other JSRD subgroups (about 30%).

Supratentorial abnormalities have been reported occasionally in OFD VI, including absence of the pituitary gland [18], migration disorders [32], occipital encephalocele [33,34], or hypothalamic hamartoma [8,19,20,30,32,35,36]. In our cohort, however, supratentorial abnormalities were present in more than two-thirds of the patients, compared with about one-third of patients in other JSRD subgroups [21]. Interestingly, in four OFD VI patients we found fused thalami, which has not been reported previously in JSRD and is a typical neuroimaging finding in holoprosencephaly [37]. The relevance of hypothalamic hamartomas in OFD VI will be discussed below.

### Neurological and cognitive outcome

In OFD VI, not only the neuroimaging pattern but also the impairment of neurological and cognitive outcome is more severe than in other JSRD subgroups. Indeed, about 50% of our patients did not learn to walk and about 70% did not develop intelligible speech, compared with only 12-33% and 27% respectively [38,39]. In other JSRD subgroups epilepsy is rare in the absence of associated migration abnormalities [4]. Three patients in our study had epileptic seizures and only one had a migration disorder, suggesting that a higher incidence of epilepsy may also be part of the more severe neurological phenotype.

Developmental delay and/or cognitive impairment of variable degrees are present in almost all patients with JSRD [5,40] and a normal full scale IQ was only reported in two exceptional patients [5,41]. In OFD VI, developmental delay and/or cognitive impairment were considered to be key features in the original report [17]. Four patients were shown to have a developmental quotient of 26-50 [18,19,30] and a full scale IQ of 46 was reported in another patient [32]. Normal cognitive functions (without formal IQ assessment) have only been reported in one patient, attending a regular school [30]. In our series 14 of 15 patients had abnormal cognitive development or functions, thus matching the previous reports. In the majority of the patients the profound degree of learning disabilities did not allow us to perform a neuropsychological examination. Formal IQ assessment was only possible in three patients. Two had moderate cognitive impairment (full scale IQs of 58 and 47), while cognitive functions were within normal limits in only one patient (full scale IQ of 82). The frequency of cognitive impairment and the rarity of a normal IQ are similar in OFD VI and other JSRD subgroups. The level of cognitive impairment, however, appears to differ: in this study, 64% of the patients had a profound cognitive impairment with almost absent cognitive development compared with 30% in other JSRD subgroups [38]. The higher severity of both neuroimaging pattern and impairment of neurological and cognitive outcome in OFD VI compared with other JSRD subgroups might be explained by a causal correlation between these findings.

### Involvement of other organs

In previous reports, involvement of eyes [8,42] or kidneys [43,44] has only been reported in four OFD VI patients. In our cohort, involvement of other organs was more common and present in 69% of the patients. Ocular involvement, mostly as a coloboma, was present in 50% of the patients, whereas our findings confirm that renal involvement is rarely associated with OFD VI and liver involvement is not a feature of OFD VI. Due to the young median age of the present patient cohort renal and/or liver involvement may still develop and no firm conclusions can be drawn at present. Interestingly, one patient of our cohort had Hirschsprung disease, which has also been described in one patient with "pure" JS [45] and in one patient with Bardet-Biedl syndrome, another ciliopathy [46]. This association does not seem to be coincidental and cilia have been implicated recently in neural crest development [47].

### Oral findings

Oral findings were considered as essential clinical hallmarks for OFD VI [17,30,32,36,44,48]. Single or multiple tongue hamartomas represent the most common form. In our cohort, however, three patients did not have tongue hamartomas, but had other oral signs such as multiple frenula or cleft palate. Multiple oral frenula are specific for OFD VI, whereas cleft lip and/or palate have been reported in patients within other JSRD subgroups [2-4]. Three patients in our study had an upper lip notch, which has been previously reported in two patients with OFD VI [49,50].

It is arguable whether oral findings are an absolute criterion for OFD VI. A child with MTS, bilateral mesoaxial hand-polydactyly, and bilateral big-toe-duplication, but without oral findings, has been reported [34]. These neuroimaging and limb findings are highly suggestive of OFD VI.

In the single affected male of the second family reported by Coene et al., JBTS10 and mutation in the *OFD1 *gene, an upper lip notch, a deep midline groove of the tongue and a postaxial polydactyly were described [51]. He did not have involvement of other organs. His MRI revealed the MTS, a thickened midbrain and a foramen magnum cephalocele. Based on the oral findings (upper lip notch), this patient would full-fill the diagnostic criteria for OFD VI (as discussed below). In the eight male patients of the other family with JBTS10 and mutations in the *OFD1 *gene, however, no oral findings, but only postaxial polydactyly in 3 were reported [51].

### Polydactyly

Polydactyly has been reported in several patients with JSRD [52,53]. The most common form is represented by postaxial polydactyly variably affecting hands and feet. In OFD VI, however, polydactyly is present in almost all patients and its most characteristic forms are a mesoaxial hand polydactyly arising from an additional central metacarpal bone or from a bifid or Y-shaped third metacarpal bone [17,30,35], and a preaxial foot polysyndactyly with bifid hallux [17,30]. Mesoaxial hand polydactyly is extremely rare and specific for OFD VI among the JSRD phenotypes, but not consistent in OFD VI: different forms of polydactyly have been previously reported and also found in this study [17,18,30,44,54].

Patients with mutation in the *KIF7 *gene in the context of an acrocallosal or fetal hydrolethalus phenotype were frequently found to have polydactyly and occasionally cleft palate, but not mesoaxial polydactyly, tongue hamartoma, additional frenula, or hypothalamic hamartoma [55]. Postaxial polydactyly has been also described in patients with JBTS10 and mutation in the *OFD1 *gene as discussed above [51].

### Hypothalamic hamartomas

Hypothalamic hamartomas are also characteristic of OFD VI and have not been reported in other JSRD phenotypes. However, they are not consistent and pathognomonic for OFD VI. The majority of hypothalamic hamartomas are sporadic and approximately 5% are associated with the diagnosis of Pallister-Hall syndrome (PHS, OMIM 146510) [56-59]. Patients with PHS and mutations in the *GLI3 *gene may have also clinical manifestations that overlap the OFD VI-phenotype, namely mesoaxial or postaxial polydactyly and oral findings including additional frenula, tongue hamartomas, and cleft palate [60,61]. In PHS patients with a *GLI3 *mutation however, the MTS has never been reported, and its presence allows the differentiation of OFD VI from PHS. Johnston et al. reported two patients with presumed PHS, a phenotype overlapping OFD, and a MTS [60], and Avila et al. reported also a similar patient [61]; however, none of these patients carried mutations in the GLI3 gene. Therefore it remains uncertain whether these patients have PHS or in fact may be examples of OFD VI. Furthermore, PHS is inherited in an autosomal dominant manner and individuals with PHS may have an affected parent or may have the disorder as the result of a de novo mutation [59]. On the contrary, OFD VI is inherited in an autosomal recessive manner.

Hypothalamic hamartomas have also been described in the holoprosencephaly polydactyly syndrome, Smith-Lemli-Opitz syndrome type 2, hydrolethalus syndrome, and cerebro-acro-visceral early lethality (CAVE) multiplex syndrome [62]. Finally, they have been recently found in the short rib-polydactyly syndrome, another group of disorders associated with mutations in ciliary genes (Palma-Dias R, personal communication at the conference on Fetal MRI, Vienna 2010). Additionally, hypothalamic hamartomas should be distinguished from interpeduncular heterotopias on the basis of their locations [63]. Interpeduncular heterotopias were initially reported in three patients with "pure" JS, but we have also detected them in two OFD VI patients.

### Diagnostic criteria

Based on the literature and the present study, we consider the presence of MTS mandatory to diagnose OFD VI. MTS has not been described in any other type of oral-facial-digital syndrome and its presence allows the differentiation of OFD VI from other types.

The oral findings that are present in OFD VI and differentiate OFD VI from the other types of JSRD are tongue hamartomas, multiple frenula, and a midline notch of the upper lip. Clefting of the upper lip or palate occurs in OFD VI but has also been reported in other JSRD subgroups and does not count as a differentiating sign.

The characteristic limb manifestation of OFD VI is mesoaxial polydactyly, which does not occur in any other type of JSRD. Preaxial and postaxial polydactyly occur both in OFD VI and other types of JSRD and therefore are not differentiating signs.

Hypothalamic hamartomas are specific for OFD VI as they have not been reported in any other JSRD type.

Neither MTS, the oral findings, mesoaxial polydactyly, nor hypothalamic hamartoma are pathognomonic as each can occur in other entities as well. However, the present study shows that the combination of MTS with one (or more) of the three other signs is pathognomonic for the diagnosis of OFD VI. Therefore we suggest that these form the diagnostic criteria for OFD VI (Table 4). We realize that no patient with MTS and hypothalamic hamartoma but without oral or limb defects has been described to date, but in theory can occur and then our criteria would be sufficient for the diagnosis of OFD VI. We are aware that the suggested diagnostic criteria do not match the inclusion criteria for this study. We derived the inclusion criteria from the literature while the diagnostic criteria are based on the literature and the present study. Indeed both patient 4 and the siblings patients 12 and 13 do not fulfill the diagnostic criteria suggested here (although the siblings fulfill the criteria together).

**Table 4:** Diagnostic criteria for OFD VI


Molar tooth sign (MTS)* *and *one *or *more of	1. Tongue hamartoma(s) *and/or *additional frenula *and/or *upper lip notch
	
	2. Mesoaxial polydactyly of one or more hands or feet
	
	3. Hypothalamic hamartoma

*, MTS is required for the index patient of a family; if MRI is not available, affected relatives need one of 1-3

The presence or absence of the other findings that can be present in OFD VI, such as cognitive impairment, ocular colobomas, and duplicated halluces, does not affect the diagnosis of OFD VI.

### Genetic findings

In two OFD VI patients, including one fetus, a homozygous mutation in the *TMEM216 *gene was found [28]. However, we failed to identify *TMEM216 *mutations in 12 patients from our cohort. Sequencing analysis of the *KIF7 *gene in six OFD VI patients revealed only one heterozygous missense mutation, which was also detected in two patients with Bardet-Biedl syndrome (each one also carrying another heterozygous mutation in a *BBS *gene), as well as in 3 out of 384 controls [55]. One isolated male patient with JBTS10 and a frameshift mutation, p.E923KfsX3, in exon 21 of the *OFD1 *gene fulfilled our diagnostic criteria for OFD VI [51]. However, eight male patients with JBTS10 and mutations in the same gene did not match our suggested diagnostic criteria [51], and several female patients with OFD VI have been reported, excluding a predominant X-linked recessive inheritance. From these preliminary findings, it appears that neither *TMEM216 *nor *KIF7 *or *OFD1 *represent major genes causative of the OFD VI phenotype, and the genetic basis of this condition still remains elusive.

We also found a heterozygous mutation in the *AHI1 *gene in the two OFD VI twins included in this study. This heterozygous mutation, as well as the heterozygous mutation found in the *KIF7 *gene, are likely to represent genetic modifiers of the phenotype. Such modifiers have previously been reported in JSRD and other ciliopathies and may explain the phenotypical variability associated with mutations in the same gene [64,65]. One example was the identification of heterozygous changes in the *AHI1 *or *CEP290 *genes in individuals with homozygous *NPHP1 *deletions, which may lead to a more severe neurological phenotype [66]. In conclusion, to date no major gene has been consistently associated with OFD VI and the mutations in the *TMEM216 *gene remain occasional. In siblings 12 and 13, with consanguineous parents, genome-wide homozygosity mapping allowed all known gene loci to be excluded, suggesting the existence of a novel gene causative of OFD VI. Whole exome analysis is currently being carried out in this family. Should this approach be successful, mutation analysis in a larger cohort of OFD VI cases will reveal whether the newly identified gene represents a major determinant for this specific JSRD phenotype.

### Limitations

We are aware of some limitations in our study. Although this is the largest reported cohort, the number of OFD VI patients is still small. Additionally, not all JSRD genes were sequenced in all patients. Moreover, the neuroimaging evaluation includes only qualitative, not quantitative measurements. Finally, a formal neurocognitive evaluation was only performed in a minority of the patients.

## Conclusions

This study confirms that the neuroimaging pattern and the impairment of neurological and cognitive outcome are more severe in OFD VI than in other JSRD subgroups. We suggest there is a correlation between the more severe neuroimaging findings and the more pronounced neurological phenotype/cognitive impairment in OFD VI. However, this observation will remain speculative until larger series of patients are reported.

The diagnosis of OFD VI is still currently based on clinical findings and neuroimaging [67]. In view of the literature and our cohort, we suggest as diagnostic criteria for OFD VI: MTS and one or more of the following: 1) tongue hamartoma(s) and/or additional frenula and/or upper lip notch; 2) mesoaxial polydactyly of one or more hands or feet; 3) hypothalamic hamartoma. These criteria allow the diagnosis to be made even in the absence of oral findings and/or polydactyly. The validity of these criteria needs to be reassessed in additional cohorts of patients and after the identification of major genetic determinants of OFD VI.

## Abbreviations

JS: Joubert syndrome; JSRD: Joubert syndrome and related disorders; MTS: molar tooth sign; OFD VI: Oral-facial-digital syndrome type VI; PHS: Pallister-Hall syndrome; SCP: superior cerebellar peduncles

## Competing interests

The authors declare that they have no competing interests.

## Authors' contributions

EB, EMV, and AP conceptualized and designed the study; EB and EMV generally supervised the study; EB, AP, EMV, GV, and RCMH analyzed and interpreted the data; AP drafted the manuscript; EB, EMV, and RCMH critically revised the manuscript for intellectual content; all authors participated in the acquisition of data and read and approved the final manuscript.
